# Transcriptome analyses suggest that changes in fungal endophyte lifestyle could be involved in grapevine bud necrosis

**DOI:** 10.1038/s41598-020-66500-0

**Published:** 2020-06-11

**Authors:** Thales Henrique Cherubino Ribeiro, Christiane Noronha Fernandes-Brum, Claudia Rita de Souza, Frederico Alcantara Novelli Dias, Osmar de Almeida-Junior, Murilo de Albuquerque Regina, Kellen Kauanne Pimenta de Oliveira, Gabriel Lasmar dos Reis, Larissa Maia Oliveira, Fernanda de Paula Fernandes, Laurent Torregrosa, Jorge Teodoro de Souza, Antonio Chalfun-Junior

**Affiliations:** 1 Laboratory of Molecular Plant Physiology, Department of Biology, Federal University of Lavras, Lavras, Brazil; 2 Technological Center of Grape and Wine Research, Agronomical Research Institute of Minas Gerais, Caldas, Brazil; 3 Coordination of Integral Technical Assistance of São Paulo, São Paulo, Brazil; 4 Department of Phytopathology, Federal University of Lavras, Lavras, Brazil; 5 Montpellier SupAgro, Montpellier, France

**Keywords:** Plant molecular biology, Plant symbiosis

## Abstract

Bud necrosis (BN) is a common disorder that affects *Vitis vinifera* L. and reduces its potential yield. To minimize the losses caused by BN, the double pruning management was applied in Brazilian Southeast vineyards. In this management strategy plants are pruned at the winter to promote a vegetative cycle and then, at summer, to promote the reproductive cycle at optimal environmental conditions. To investigate the relationship of BN and the double pruning management RNA-seq libraries were sequenced from healthy and necrotic tissues at four different stages of the year. The comparison of differentially expressed genes in necrotic and non-necrotic tissues showed an enhanced expression of genes related to cell death possibly induced by endophytic microorganisms in the necrotic tissues. The *de novo* assembly, characterization and quantification of transcripts within the RNA-seq libraries showed that genes from the endophytic fungus *Alternaria alternata*, responsible for the production of toxic compounds were highly expressed under BN. Here we propose a model in which unfavorable conditions and reduced carbohydrate levels in buds can promote the switch from a biotrophic lifestyle to a necrotrophic lifestyle in the endophytic fungi, which seems to be involved in the development of BN.

## Introduction

Grapevine (*Vitis vinifera* L.) is an important horticultural crop with a large variety of derived products. Its berries can be consumed as fresh or dried fruits and also used for the production of juices, liquors and wines. One of the main factors affecting grape production worldwide is the occurrence of a disorder known as primary Bud Necrosis (BN)^1,2^. This disorder is widespread and has been reported to occur in Australia^2^, United States^3^, Japan^4^, Iran^5^, Europe^6^ and Brazil^7^ leading to reduction of yield potential.

Primary BN is characterized by the death of the primary buds, considered to be the most productive. Secondary and tertiary buds often derive shoots believed to be less fruitful and with smaller clusters^2^. BN is difficult to be diagnosed once buds may appear healthy externally for a long time and at this condition, a dissection analysis of the buds under an stereomicroscope can detect it^2^. Under primary BN, photoassimilates will be diverted to the less fruitful buds^8^.

The causes of BN in grapevines, including the necrosis of the primary, secondary and tertiary buds, are still unknown. Its incidence can vary according to the vineyard, location, year and cultivar^2^. Many explanations are being proposed to be the cause of this phenomenon including high shoot vigor^2,8–11^, influence of gibberellins^4^, high temperature^1^, vigor of the rootstocks^1^, canopy shading^3,12^ and reduced carbohydrate levels^1,9^. Even low temperature might be attributed as the cause^13^. However, it is still unknown if, and how, all these factors interact to promote BD in different environmental conditions.

In the last available Food and Agriculture Organization report, in 2017 the world grape yield was 74 million tons and Brazil was responsible for 1.9 million ton (http://www.fao.org/). Approximately 33% of Brazilian grape production is represented by wine grapes. The majority of the Brazilian territory does not have climatic characteristics that are favorable for wine grapes, which requires high thermal amplitudes with cold nights and low precipitation^13^.

In Brazil, the regions where wine grape production is more intensive are the South and Southeast. However, environmental factors like the high precipitation levels during summer cause a negative effect in grape production once high humidity favors the occurrence of fungal diseases^14^. Under these subtropical conditions, the commercial grape production is completely unavailable due to unfruitful shoots induced by the change of annual yield pruning from winter to summer season^13^. For this reason, wine grape growers in the Southeast have adopted the double pruning management to shift the harvest from the wet summer to the autumn-winter months, when the low rainfall and high temperature amplitude are suitable for the accumulation of sugars and synthesis of phenolic compounds, ultimately leading to increased yields^15–17^ as well as avoiding fungal proliferation. This strategy consists in two pruning events, the first at the winter (August-September) to promote the non-productive cycle and the second pruning, which is performed during the summer (January or February) to promote the reproductive cycle^15^.

The double pruning management resulted in two growth cycles and one harvest per year. Although it has greatly improved the wine grape quality, it has also increased the production costs^13^. The adoption of an annual single pruning in the summer (January) to avoid the extra costs and induce the harvest in the winter months resulted in completely unfruitful buds in Syrah grapevines cultivated in the Southwest of Brazil. This unfruitfulness was recently attributed to the occurrence of BN in primary and secondary axes of latent buds^13^. These authors also observed that latent buds developed during productive cycles showed higher rates (above 80%) of BN incidence as compared to BN rates in latent buds developed during the non-productive cycles (less than 20%).

A clear positive relationship between more severe pruning treatments and higher BN incidence was reported by Collins and Rawnsley^2^. In these cases, the overall number of buds that grew after pruning was higher than less severe pruning methodologies^2^. Under these circumstances, the competition of different sink organs (increased number of developing buds) for photoassimilates can imbalance source/sink dynamics and, aligned with unfavorable environmental conditions, can ultimately activate programmed cell death (PCD) pathways^2,9,18–20^.

Endophytes may comprise species that colonize plants without causing any disease and also latent pathogens that eventually change their lifestyle from biotrophic to necrotrophic, thereby inducing disease symptoms in host plants^21^. Endophytic fungi were not previously investigated in connection with bud necrosis. Therefore, transcriptomics coupled with bioinformatic analyses were used to investigate BN in a Syrah vineyard under double pruning management and its relationship with PCD.

Transcriptomic analyses revealed a higher expression of genes from the endophytic fungi in necrotic as compared to non-necrotic buds in both growth seasons. Indeed, we have showed that endophytic fungi were present in both necrotic and non-necrotic grapevine buds by isolation and morphological characterization. Complementarily, grapevine genes involved in response to endophytic fungi and PCD are comparatively more expressed in necrotic tissues. Therefore, our hypothesis is that not only physiological and climatic factors are affecting BN but also the interaction with *Alternaria alternata* and, potentially, other endophytic fungi.

## Methods

### Plant material

Buds of *Vitis vinifera* were collected in 4 months (March, April, November and December), in 2015, from a Syrah variety commercial vineyard in the municipality of Três Corações, Minas Gerais State, Brazil (21°41′ S, 45°15′ W). Plants were submitted to the double pruning method to induce harvesting in the winter. By doing so, plants were pruned at the beginning of January (the productive cycle) and then at the beginning of August (the non-productive cycle). During the non-productive cycle, all grape clusters were removed. Plant material was collected in biological triplicates of four conditions; in March (No Necrosis 1 - NN1), April (Necrosis 1 – NE1), November (No Necrosis 2 - NN2) and December (Necrosis 2 - NE2). The samples were immediately kept in liquid Nitrogen (N_2_) until storage in deep Freezer at −80 °C. Each library was constituted by a pool of 54 buds from 3 branches of 3 plants.

### RNA extraction for mRNA sequencing

The buds were ground in liquid N_2_ with mortar and pestle to form a fine powder. Afterwards, the RNA extraction was performed with the CTAB method developed by Chang *et al*.^22^. Then, the RNA was treated with DNAse (*Turbo DNAfree* - Ambion) following the manufacturer’s instructions and then sent to a third-party facility (Genewiz, South Pleinfield, NJ, USA) for sequencing. The sequencing libraries were made with poli-A tail selection and single-end reads were sequenced with Illumina HiSeq. 2500 - Rapid Run, V2 chemistry. Sequenced libraries were submitted to Sequence Read Archive under Bioproject number PRJNA558999.

### Differential expression analysis with *Vitis vinifera* reference genome

Approximately 173 million single-end reads were sequenced. After quality analyses, adaptors and low-quality reads removal were performed with Trimmomatic v. 0.36^23^. Nearly 167 million reads were used to the alignment against the *Vitis vinifera* genome v12 available at https://phytozome.jgi.doe.gov/ ^24^ with STAR v. 2.5.3a^25^. About 82% of the reads were uniquely mapped to the reference genome. Afterwards, the alignment files were ordered with Samtools 1.6 and the quantification of the mapped reads to exons was performed with the htseq-count script^26^.

The identification of genes Differentially Expressed (DE) among conditions was performed with the *edgeR* package^27^ in the *R* statistical environment^28^. The expression value of each gene in a given condition was fitted with a Generalized Linear Model^29^. Genes were considered DE if they were at least two times more expressed in a given condition (−1 < Log2FC > 1) and FDR (False Discovery Rate) values below 0.05^30^. All the DE genes were submitted to the SEA (Singular Enrichment Analysis) with the online tool *Agrigo*^31^ available at http://bioinfo.cau.edu.cn/agriGO/analysis.php.

#### Starch and sucrose metabolic pathway DE genes inference

Genes found to be DE with ontology terms related to the enriched biological process of starch catabolism (GO:0005983) were used as query to the identification of key enzyme coding genes responsible for the starch breakage into reducing sugars. Those DE genes were mapped to enzymes in the Kyoto Encyclopedia of Genes and Genomes (KEGG - available at https://www.kegg.jp) database with the BlastKOALA tool^32^.

### *De novo* assembly of the meta-transcriptome and differential expression

The identification of transcripts, not necessarily belonging to *Vitis vinifera*, was done by the *de novo* assembly of the quality controlled reads from all libraries with Trinity v. 2.3.2^33^ and potential protein encoding inference was performed with TransDecoder v. 5.3.0^34^. Afterwards, the resulting predicted sequences were annotated by homology search against the protein sequences deposited at the non-redundant (nr) database of the NCBI (National Center for Biotechnology Information) until July 2017 with Blast2GO^35^. The identification of the DE genes of the meta-transcriptome was performed by RSEM^33^ quantification and *edgeR*^27^ analysis using the scripts available within the Trinity package. In this approach, a gene was considered DE if the difference in the expression level in two conditions were at least 4 times higher in one of them (−2 < Log2FC > 2) and FDR below 0.05.

### Bud necrosis incidence assessment

The proportion of necrotic buds was evaluated in the same months in which samples were collected for RNA extraction; March, April, November and December of 2015. In each month 9 plants were randomly selected and 10 buds from a productive branch of each plant were removed to be dissected and analyzed under a stereomicroscope. In this way, a total of 90 buds were observed for each condition and the darkened ones were considered necrotic while all others were considered healthy.

### Isolation and identification of endophytic fungi from vine buds

Vine buds were collected from a Syrah germplasm in the experimental station of EPAMIG (Empresa de Pesquisa Agropecuária de Minas Gerais), Caldas (22°55′S, 46°23′W) in January 2019. These isolations were performed four years after the beginning of the study and were done to confirm the presence of the endophytes that were detected in the transcriptomic analysis. Compound buds at node positions 1 to 5 from 24 vine shoots were used in the isolations by dissecting under a stereomicroscope. The primary buds were excised from the shoots and classified into necrotic or healthy. Necrotic and healthy buds were separately surface-sterilized by immersion in 70% ethanol for 3 min followed by immersion in NaOCl 1% for 5 min, washed three times in sterile distilled water and plated on 10% potato dextrose agar (PDA). Additionally, samples of approximately 5 mm from the bark, shoot cortex and vascular cylinder were surface-sterilized and plated as described above. The water from the last rinse of each of the examined tissues was plated to check for contaminants and only plates without any contaminant growth were used in the identifications. The plates were incubated at 25 °C for two weeks and the fungi growing from the buds were transferred to fresh 10% PDA plates for identification. Morphological fungal identification at the genus level was done by microscopical observations of the spores produced on plates. Fungi that did not produce spores could not be identified.

## Results

### *Vitis vinifera’s* genes expressed in necrotic buds differ from the ones expressed in non-necrotic buds

**Figure 1:**
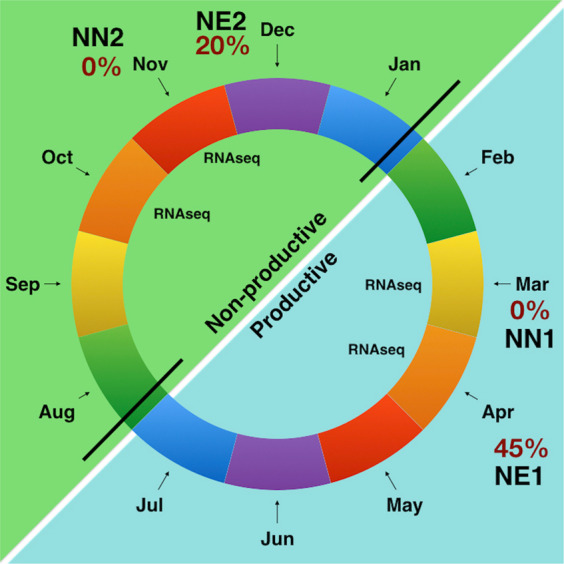
The double pruning schematic representation. The black lines in the months of January and August indicate the pruning. The percentages of bud necrosis incidence are indicated in red at the collecting material points. Both the productive and non-productive cycles are shown for the year 2015, in cyan and green, respectively.

In the first semester of 2015 the BN incidence during the productive cycle in March was 0% (No Necrosis 1 - NN1). On the other hand, the BN incidence in April of the same productive cycle increased to 45% (Necrosis 1 – NE1). In the second semester of the year, during the non-productive cycle, BN incidence was 0% during November (No Necrosis 2 - NN2) and 20% in December (Necrosis 2 - NE2), Fig. 1.

**Figure 2:**
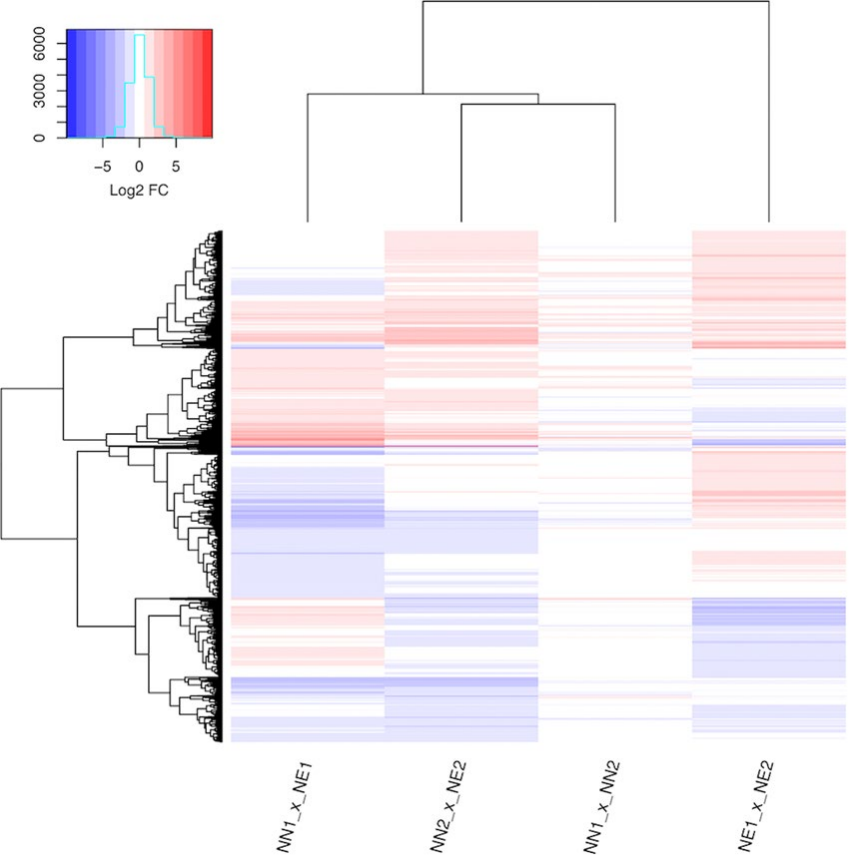
Heatmap with all DE genes. Each column represents a comparison between two conditions whereas each line represent a DE gene across the underlying comparison. Both comparisons and genes are clustered based on their expression similarities. The up-regulated genes are colored in red hues and the down-regulated genes are colored in blue hues. Values are presented as logarithmic base 2 of fold change.

From the 26,346 genes in the *Vitis vinifera* reference genome (Genoscope 12x), 3,895 were Differentially Expressed (DE) - approximately 15% - in all of the compared conditions (Fig. 2). Many of these genes (1,025) presented a similar expression profile in the comparisons between necrotic and non-necrotic conditions in both semesters (Fig. 2, Supplementary Tables 1 and 2). We also evaluated the expression profiles of genes in the background comparisons, the genes that were DE in non-necrotic versus non-necrotic in both semesters and the ones DE in necrotic versus necrotic in both semesters (Fig. 2, Supplementary Tables 3 and 4 respectively). Only 61 genes were DE when comparing the non-necrotic stages (NN1 x NN2). On the other hand, 2,013 genes were DE when comparing the necrotic stages (NE1 x NE2), with 673 exclusively DE in this comparison.

**Figure 3:**
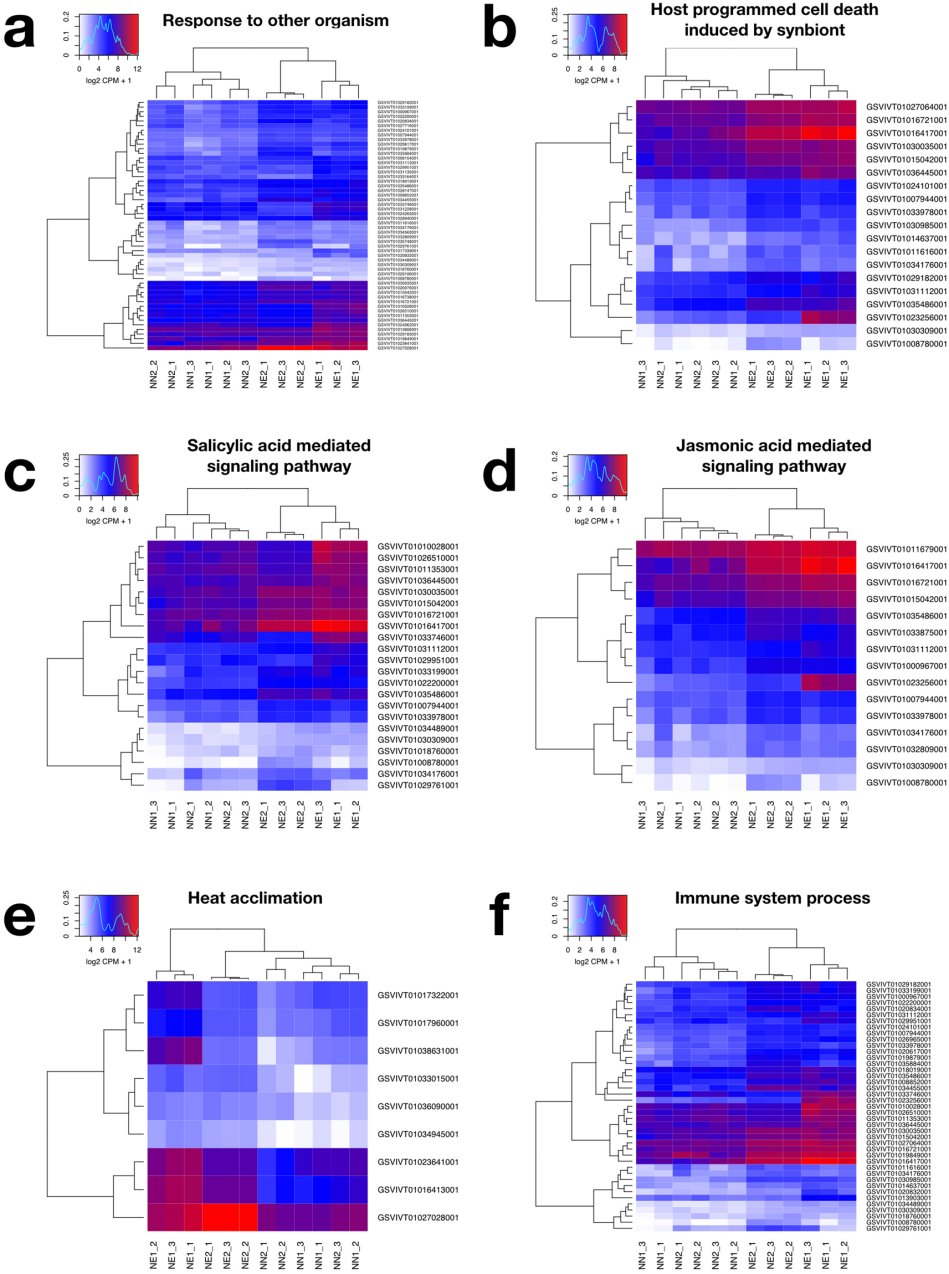
Part of the Gene Ontology enrichment results in a heatmap representation. Each column of a given heatmap represents a biological repetition of a given condition and each line represents a DE gene of given gene ontology classification. Expression values are presented as logarithmic base 2 of normalized Counts Per Million (CPM) across samples plus 1. Highly expressed genes are colored in red hues whereas lower expressed genes are colored in blue. (**a**) Response to other organism (GO:0051707); (**b**) host programmed cell death induced by symbiont (GO:0034050); (**c**) salicylic acid mediated signaling pathway (GO:0009863); (**d**) jasmonic acid mediated signaling pathway (GO:0009867); (**e**) response to oxidative stress (GO:0006979), and (**f**) immune system process (GO:0002376).

The most relevant comparisons for this study were those contrasting the necrotic and non-necrotic conditions within the same cycle, i.e. NN1 x NE1 and NN2 x NE2. We found 78 enriched Gene Ontology (GO) terms shared by the up-regulated genes in both of the necrotic conditions. They included biological processes such response to other organism (GO:0051707), host programmed cell death induced by symbiont (GO:0034050), salicylic acid mediated signaling pathway (GO:0009863), jasmonic acid mediated signaling pathway (GO:0009867), response to oxidative stress (GO:0006979), immune system process (GO:0002376), positive regulation of flavonoid biosynthetic process (GO:0009963) and others (Fig. 3, Supplementary Tables 5 and 6 and Supplementary Figs. 1 to 6).

### Starch catabolic process genes are up-regulated during necrosis in the first semester

**Figure 4:**
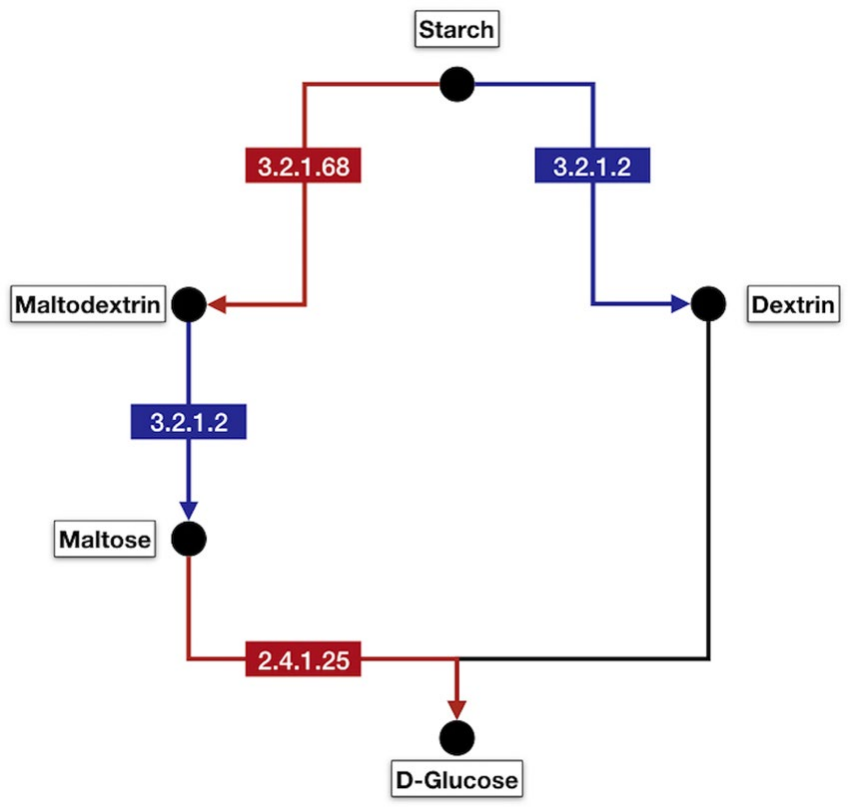
Starch and sucrose metabolism pathway highlighting enzyme coding genes related to starch catabolism in the necrotic conditions (NE1 and NE2). Blue boxes represent beta amylase (EC:3.2.1.2) which is DE in both of the necrotic conditions. Red boxes are the enzymes 4-alpha-glucanotransferase (EC:2.4.1.25) and isoamylase (EC:3.2.1.68) that are DE only in the necrotic condition of the first semester.

A total of 8 genes responsible for starch catabolic processes (Biological Process GO:0005983) are up-regulated in the necrotic condition during the first semester when compared to the non-necrotic condition of the same period (NN1 x NE1) (Fig. 1, Supplementary Fig. 3). This ontology term was not enriched for DE genes in the comparison of necrotic versus non-necrotic in the second semester (NN2 × NE2) when the BN incidence was lower (Fig. 1, Supplementary Fig. 6). One gene (GSVIVT01013272001) related to starch catabolism was up-regulated in both of the necrotic conditions (NE1 and NE2). This gene encodes a beta amylase enzyme (EC:3.2.1.2) responsible for the removal of successive maltose units from the non-reducing ends of the starch or maltodextrin chains (Fig. 4, blue boxes)^36^.

Among the up-regulated genes encoding for enzymes related to starch catabolism exclusively in the NE1 are two 4-alpha-glucanotransferases (EC:2.4.1.25 - GSVIVT01025810001 and GSVIVT01022223001) and one isoamylase (EC:3.2.1.68 - GSVIVT01009087001), which are shown in the red boxes of Fig. 4. In this way we can expect a higher conversion of starch to maltose and, subsequently to D-glucose in the first semester in comparison with the second semester.

### Endophytic fungi are present in both necrotic and non-necrotic buds

Ontology terms such as response to other organism (GO:0051707) and response to fungus (GO:0009620) suggested the presence of endophytic fungi in the transcriptome. To confirm the presence of these endophytes in *Vitis vinifera* and discard the hypothesis of eventual contamination during sequencing library preparation, we collected additional samples to perform the identification of the fungal species by observing their spores and fruiting bodies with an optical microscope. Doing so, we found that *Alternaria alternata* and other endophytic fungi were present in buds of *Vitis* in both healthy and necrotic buds (Table 1).

**Table 1:** Percentage of the sampled fragments colonized by endophytic fungi.

Fungi	Healthy buds	Necrotic buds	Bark	Cortex	Vascular cylinder
*Alternaria*	**46.2**	**44.4**	**0.0**	**0.0**	**0.0**
*Pestalotiopsis*	**43.1**	**38.9**	**6.7**	**13.3**	**13.3**
Yeasts	**0.0**	**0.0**	**20.0**	**6.7**	**13.3**
*Colletotrichum*	**3.1**	**0.0**	**0.0**	**0.0**	**0.0**
*Epicoccum*	**13.8**	**0.0**	**0.0**	**0.0**	**0.0**
Dark fruit bodies^a^	**9.2**	**16.7**	**0.0**	**0.0**	**0.0**
DS no fruit^b^	**7.7**	**14.8**	**0.0**	**0.0**	**0.0**

The number of fragments plated on PDA were 54 necrotic, 65 healthy buds, and 15 of each bark, cortex and vascular cylinder. The fungi were identified by microscopical observation of their spores and fruiting bodies. ^a^Non-identified fungus with a dark mycelium and empty fruiting bodies. ^b^Non-identified fungus with dark septate mycelium without fruiting bodies.

**Figure 5:**
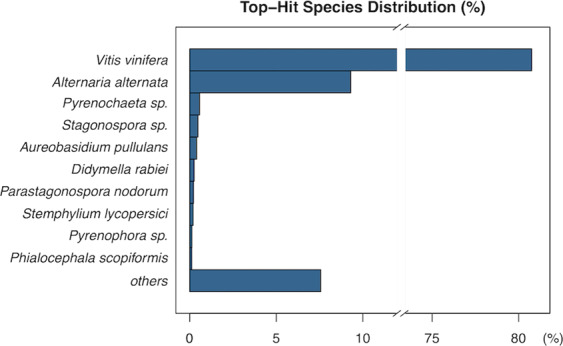
Percentage of species best-hits of the computationally assembled sequences.

The *de novo* assembly resulted in 77,680 predicted genes with a total of 144,284 isoforms. Their average contig length was 1,378.02 nucleotides and N50 value of 2,026. After potential encoding inference of the assembled sequences with TransDecoder, a total of 36,806 protein encoding genes with 86,315 isoforms was submitted to homology search with blastp^37^. Approximately 96% of the predicted protein encoding genes presented homology with proteins from the *nr* database of the NCBI^38^. Most of the isoforms (81%) had their best hit with sequences identified in *Vitis vinifera*, whereas 9% had high homology with the fungi *Alternaria alternata* and 1% with *Pyrenochaeta* sp. (Fig. 5).

**Figure 6:**
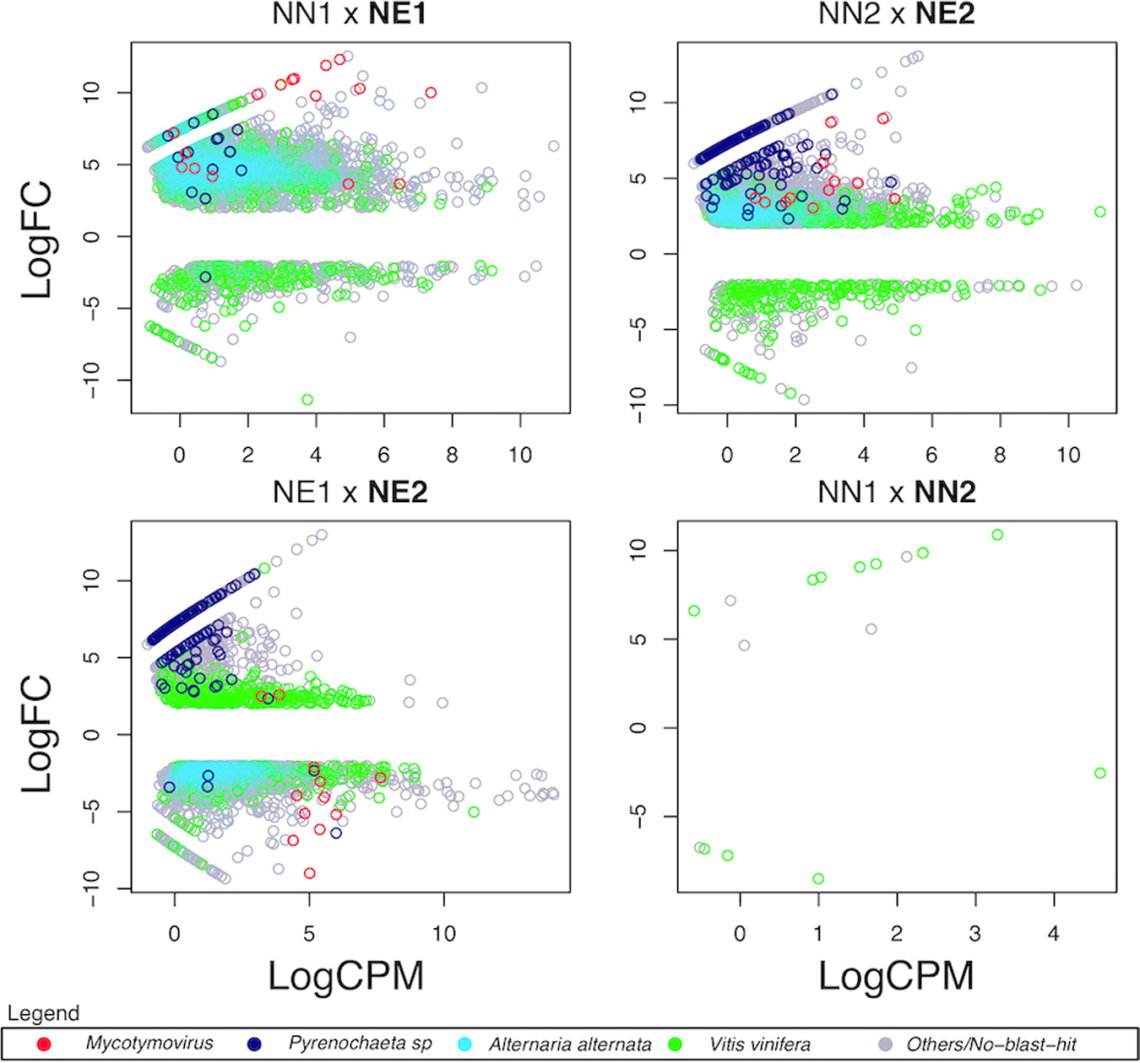
Dot plot representation of the statistically significant DE genes in the meta-transcriptome assembly. Y-axis represents the expression value in logarithmic base 2 of fold change. This metric summarizes the relative expression of a gene across the compared conditions. X-axis represents the logarithmic base 2 of the mean of Count Per Million values across all samples. This metric summarizes the confidence in the DE result. Red: *Mycotymovirus*, Dark blue: *Pyrenochaeta* sp., Light blue: *Alternaria alternata*, Green: *Vitis vinifera*, Grey: Others or without blast hit.

We also evaluated the expression profile of genes identified in the *de novo* assembly. Under the necrotic condition in the productive cycle (NE1), 6,018 genes were DE and 5,480 were more expressed compared to the non-necrotic condition NN1 at the productive cycle. Within these, 3,542 (65%) presented their best-hit with *Alternaria alternata* and mean similarity of 99%. Also, 16 viral genes (<1%) had mean similarity of 70% to a lineage similar to *Fusarium graminearum Mycotymovirus* 1; 15 genes (<1%) from *Pyrenochaeta* sp. (DS3sAY3a) with mean similarity of 97% and 442 genes (8%) from *Vitis vinifera* with mean similarity of 98% (Supplementary Table 7, Fig. 6).

Similar results were found in the second semester (non-productive cycle) when comparing NN2 × NE2 (Supplementary Table 8) where 4,440 genes were found to be DE, being 4,122 up-regulated in the necrotic condition (NE2). Within these, 1,683 (41%) presented their best-hit with *Alternaria alternata* with mean similarity of 99%, 12 genes (<1%) with *Mycotymovirus* (mean similarity of 68%), 147 genes (3%) with *Pyrenochaeta* sp. (mean similarity of 93%) and 231 genes (6%) with *Vitis vinifera* (mean similarity of 98%). Expression levels of *Pyrenochaeta* sp. (DS3sAY3a) genes were higher when compared to the first semester (Fig. 6). In striking contrast, in the non-necrotic condition in the productive cycle (NN2), from the 318 up-regulated genes, none was identified as belonging to *Alternaria alternata, Fusarium graminearum Mycotymovirus* 1 or *Pyrenochaeta* sp. (DS3sAY3a). Most of these DE genes belonged to *Vitis vinifera* (Supplementary Table 8).

In the comparison of the necrotic conditions of both semesters (NE1 x NE2) we found 1,181 genes up-regulated in the second semester and 2,360 up-regulated in the first semester (Fig. 6, Supplementary Table 9). The majority of up-regulated fungal genes in the second semester (NE2) belonged to *Pyrenochaeta* sp. (DS3sAY3a) – 81 genes (7%) with mean similarity of 93%. On the other hand, the majority of up-regulated fungal genes in the second semester (NE1) belonged to *Alternaria alternata* – 1,397 (59%) with mean similarity of 99%. Interestingly, most of the identified *Mycotymovirus* genes – 10 (<1%) mean similarity of 68% - follows the same pattern in the NE1 upregulated genes.

In the same way as the expression analysis based exclusively on *Vitis vinifera* reference genome, fewer genes – 16 - were DE when comparing the non-necrotic conditions of both semesters (NN1 x NN2), the majority of them – 11 (69%) with mean similarity of 99% - were identified as belonging to *Vitis vinifera* (Fig. 6 – Supplementary Table 10).

### Unknown Mycotymovirus genes highly expressed in necrotic buds

**Figure 7:**
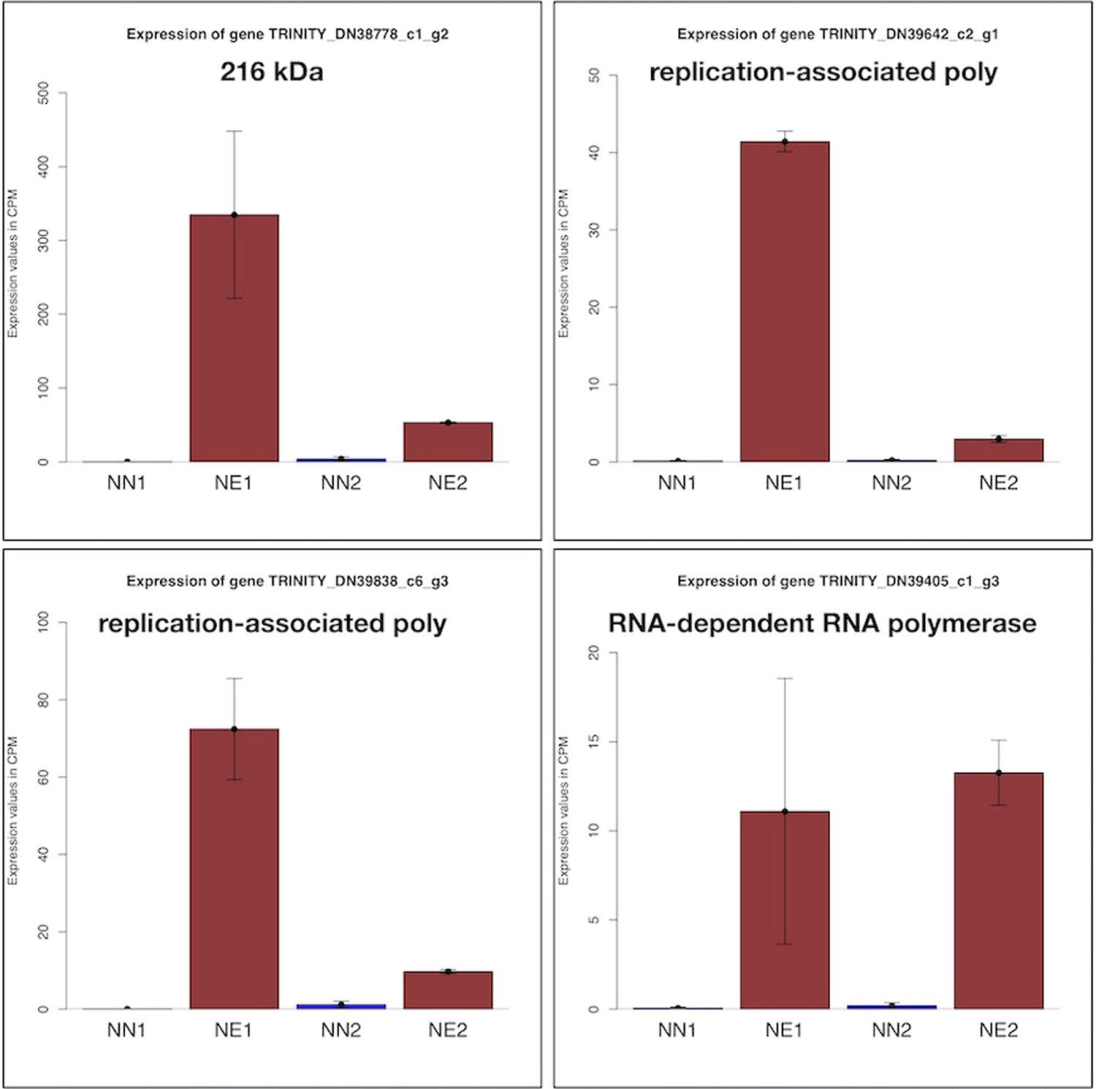
Expression of four predicted *Fusarium graminearum Mycotymovirus* 1-like protein coding genes. They are highly expressed under necrotic conditions, in special under the necrotic condition in the first semester (NE1). Brown bars represent expression values in necrotic conditions in the first and second semester of the year (NE1 and NE2) while blue bars represent expression values in non-necrotic conditions of productive and non-productive cycles (NN1 and NN2, respectively). Error Bars represent the standard error of the means. Expression values are given in normalized Counts Per Million (CPM) across the different libraries.

A total of 22 viral protein encoding genes was found in the meta-transcriptome assembly with mean similarity of 71% to a *Fusarium graminearum Mycotymovirus* 1. These sequences derived from one or more viruses that are probably using *Alternaria alternata* or *Pyrenochaeta* sp. (DS3sAY3a) as hosts. They are mainly RNA-dependent RNA polymerase replication-associated genes and present a high expression change value in necrotic conditions when compared to non-necrotic ones (Fig. 7). In the necrotic conditions (NE1 and NE2) they present a mean expression value of 29.61 Counts Per Million (CPM) while the mean expression value of all genes in the meta-transcriptome under the same conditions are 13.15 CPM after normalization by library size.

## Discussion

Grapevine bud necrosis (BN) is an important disorder responsible for decreases in grape production all over the world. The causes of BN are unknown, but several authors have shown the correlation of its occurrence with diverse physiological and environmental processes such as shoot and root vigour^8^, hormones^4^, temperature^1^, shading^12^ and carbohydrate levels^9^. In this study we show that endophytic fungi can promote BN by triggering the activation of plant’s programmed cell death (PCD) pathways.

The transcriptomic profile of DE genes in *Vitis vinifera* involved in BN process is highly complex. A total of 139 ontology terms related to biological processes, molecular functions and cellular components could be identified in the up-regulated genes during necrosis in both semesters (productive and vegetative cycles – Fig. 1), from those, 78 (56%) were shared in both of the necrotic conditions (NE1 and NE2). This result suggested that some of the molecular mechanisms underlying the necrosis are similar for the necrotic conditions in both semesters.

Despite the similarities between the enriched GO terms for up-regulated genes in the buds of necrotic conditions of both semesters, we found distinct features in their molecular machinery in different periods of the year. A total of 2,013 *Vitis vinifera* genes were identified as DE when comparing both of the necrotic conditions (NE1 and NE2). For instance, during the productive cycle (first semester), when the BN occurred in 45% of the evaluated buds, some of the most expressed genes in the NE1 condition were related to starch and polysaccharides catabolism (GO:0005983 and GO:0044247), as well as maltose metabolic process (GO:0000023) (Supplementary Table 5). On the other hand, during the non-productive cycle (second semester), genes related to chitin response (GO:0010200), response to oxygen-containing compound (GO:1901700) and respiratory burst involved in defense responses (GO:0002679) are up-regulated (Supplementary Table 6). It is possible that these differences in the metabolic responses, as well as lower expression levels of starch and polysaccharides catabolism genes during the second semester can explain the lower incidence of necrosis during this period (20% of necrotic buds). It seems that during the second semester the interaction of sugar metabolism and defense responsible genes was more adequate to deal with the environment and, therefore, programmed cell death (GO:0012501) was less intense.

The comparison of the DE genes in both of the non-necrotic conditions (NN1 and NN2) resulted in only 62 genes DE. No significant enriched GO term could be found in this comparison. This result suggests that the molecular machinery of buds in the non-necrotic conditions across both semesters works in a similar fashion despite differences in the environment.

The recurrent ontology terms in the necrotic conditions related to defense response to other organism (GO:0098542), as well as the non-alignment of nearly 18% of the reads to the *Vitis vinifera* genome allowed us to hypothesize that other organisms were present in the bud transcriptome. Towards that, the *de novo* assembly of the transcriptome was done to identify such organisms. Although the presence of bacteria was not significant in our *RNA-seq* data, it is not an evidence of its absence in buds once the sequencing libraries were made with a poli-A tail selection protocol. In this way, libraries contained mostly fragments of plant and fungal mRNA.

We showed the occurrence of endophytic fungi in *Vitis vinifera* buds grown in Brazil 4 years after the *RNA-seq* library preparation, indicating that these organisms are a permanent component of the grape microbiome. The identification of fungal species by microscopical observation of their spores and fruiting bodies confirmed the presence of at least seven endophytic fungi in grape buds (Table 1). Although the microscopically identified fungal genera did not exactly match those present in the transcriptome assembly two of the most abundant genera in the *RNA-seq* were also present in the PDA medium (*Alternaria* and possibly *Pyrenochaeta*). These changes in the endophyte population were expected once population dynamics changes in response to season, age and geographic location.

The genus *Pestalotiopsis* was identified as an endophyte by microscopical observation, however our *de novo* assembly annotation identified only 10 sequences as best-hits with *Pestalotiopsis*. This genus appears to be abundant not only in healthy and necrotic buds but also in tissues such as bark, cortex and vascular cylinder in samples collected in January 2019 (Table 1). This result suggests that this endophytic fungus does not exert a significant influence on bud necrosis. *Epicoccum* sp. and *Colletotrichum* sp. were present at relatively low densities in healthy, but not in necrotic buds (Table 1). *Colletotrichum* sp. is a common plant pathogen and *Epicoccum* sp. a beneficial fungal endophyte with biocontrol activity^39,40^. Further studies need to be performed to establish the role of this fungi in BN.

*Plasmopara viticola* is an oomycete known for causing downy mildew in vines. This pathogen affects mainly the grape crop in hot and humid regions during the vegetative and reproductive stages^41^. However, only 10 sequences had their best-hits with *Plasmopara viticola*, suggesting that this oomycete is not present or its presence is not accentuated. On the other hand, 424 genes from the fungus *Pyrenochaeta* sp. (DS3sAY3a) were identified in the meta-transcriptome. However, we could not confirm the presence of *Pyrenochaeta* with microscopical observation probably due to the lack of spore production in this species under the tested conditions. A dark fruit bodies producing fungi found living as an endophyte in buds could be *Pyrenochaeta*. Another species of this genus, *Pyrenochaeta lycopersici*, is commonly found in soil and causes necrosis in roots of *Solanum lycopersicum*^42^. These isolations were done in one season only and they were not meant to be quantitative. Their main purpose was to show the association between endophytic fungi and grape buds. To ascertain that these fungi were true endophytes we adopted a very stringent isolation protocol to ensure that these fungi were inside bud tissues Collectively, these findings suggest that BN in *Vitis vinifera* could be influenced by a change in the lifestyle of fungal endophytes.

*Alternaria alternata* is a ubiquitous fungus that can occur as a pathogenic or non-pathogenic agent in a species-specific way by a mechanism of production of host-specific toxins^43,44^. Among the susceptible species there are some varieties of apple^45^, pear^46^, tomato^47^ and some *Citrus*^48^. In *Vitis vinifera*, *A. alternata* has been reported to occur as an endophyte able to provide beneficial services to the host plant by producing substances that cause ultra-structural modifications in the mycelium of *Plasmopara viticola*, inhibiting the sporulation of the pathogenic oomycete^18^. However, the ontology term GO:0034050 (host programmed cell death induced by symbiont) enriched for up regulated genes of *Vitis vinifera* under the necrotic conditions suggests that *Alternaria alternata* can induce programed cell death in the tissues of the host plant. This hypothesis is supported by the fact that endophytic fungi can become pathogenic agents in host plants under unfavorable physiological conditions^49^.

**Figure 8:**
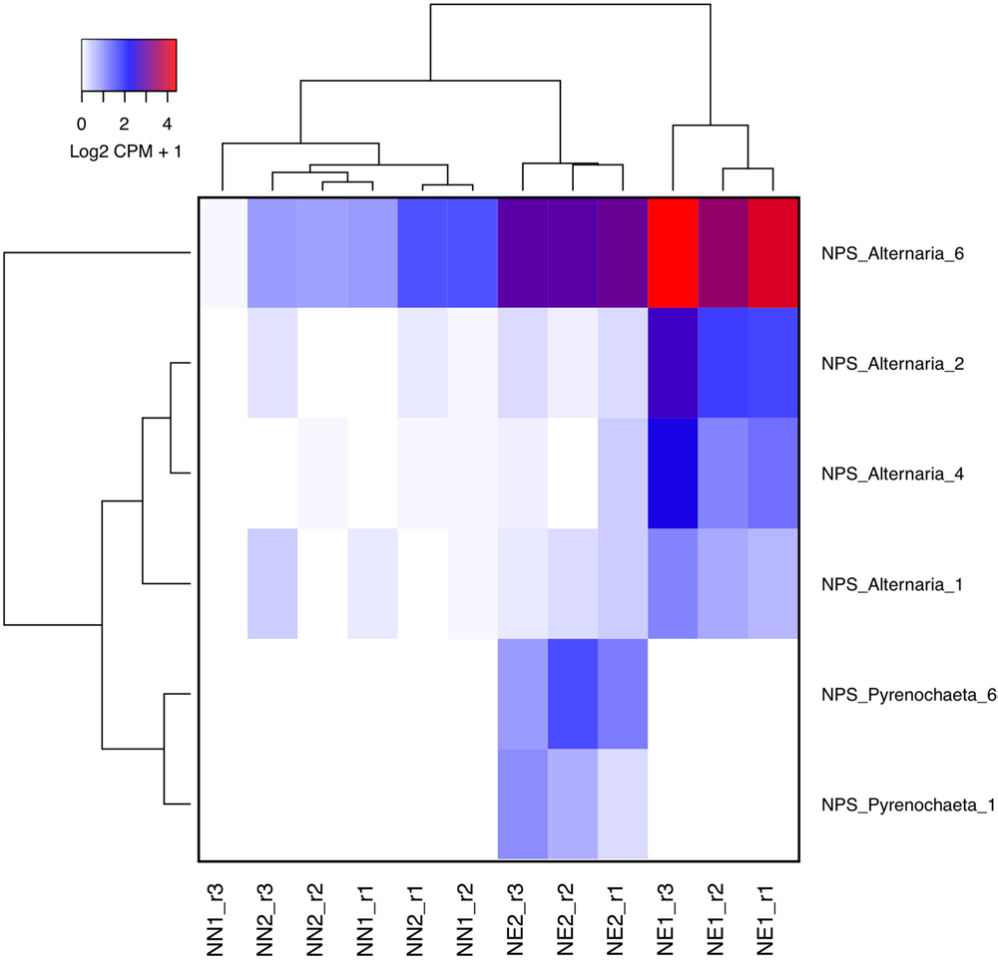
Heatmap representation of the expressed *Non-ribosomal Peptide Synthetases* (*NPS*) genes from both *Alternaria alternata* and *Pyrenochaeta* sp. In both species the *NPS6* (known as a virulence effector and to have defense activity against oxidative stress) are up-regulated in necrotic conditions. Expression values are presented as logarithmic base 2 of normalized Counts Per Million (CPM) across samples plus 1.

One of the mechanisms in which an endophytic fungus with a biotrophic lifestyle can turn into a necrotrophic is the production of non-ribosomal peptides with toxic activity by the expression of *Non-ribosomal Peptide Synthetase* (*NPS*) genes^45^. The *NPS6* may be a pathogen virulence factor as well as promote resistance to oxidative stress from hydrogen peroxide released by plants to overcome the infection^50,51^. Among the expressed *NPS* from both *Alternaria alternata* and *Pyrenochaeta* sp. the *NPS6* from *Alternaria* is the most expressed one (Fig. 8). This finding suggests that the necrotic conditions are probabilly influenced by these two endophytic fungi.

The presence of highly expressed *Fusarium graminearum* mycotymovirus 1 like genes in both of the necrotic conditions (NE1 and NE2) suggests that *Alternaria alternata* and/or *Pyrenochaeta* sp. (DS3sAY3a) are hosts for viruses. Under these circumstances the fungi may require additional sugar supply from *Vitis vinifera* to power its corrupted protein assembly and RNA-duplication machinery to be used in the replication of viruses. This process can trigger defense mechanisms, such the release of Reactive Oxygen Species (ROS), in *Vitis vinifera* that ultimately will lead to programmed cell death (GO:0012501) on the most affected bud areas. The fungal enhanced virulence by a virus was described in *Talaromyces marneffei partitivirus*-1 (TmPV1) where the virus cause modified gene expression in *Talaromyces marneffei* enhancing mycosis in mice^52^. In plants, *Alternaria alternata* chrysovirus 1 (AaCV1) was found to have two contrasting effects in the host fungus: impaired growth and increased virulence^53^.

This study provides the first cues of the involvement of endophytic fungi in grapevine BN. Although these cues are strongly supported by the transcriptomic analyses reported herein, further studies should be performed to define whether BN behaves in the same way in different cultivars, geographic locations and to confirm its relationship with endophytes.

## Conclusions

**Figure 9:**
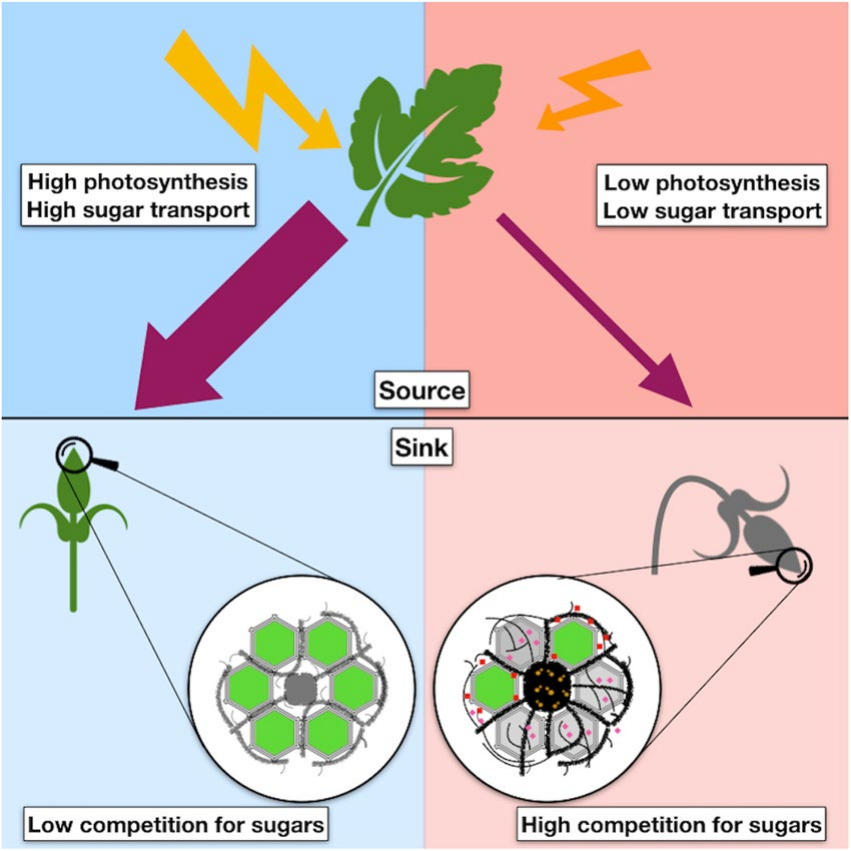
Proposed model for the metabolic interactions between grapevine and endophytic fungi that may result in bud necrosis (BN). Endophytic fungi produce filamentous mycelia and colonize biotrophically between living cells (green cells) of grapevine under optimal conditions (blue side). Alternatively, these fungal endophytes change to a necrotrophic lifestyle under stressful conditions (red side). Under optimal conditions buds are sink organs supported by photosynthetic organs (upper leaf) that can benefit from the endophytes in mutualistic relationship. Stressful events such low production of sugars from leaves (thinner purple arrow) or enhanced infection by viruses (small yellow circles) can trigger the expression of genes related to a necrotrophic lifestyle and start the synthesis of toxins (pink diamonds). Under these circumstances, necrosis (gray bud) can arise as an innate plant defense mechanism caused by the expression of programmed cell death genes and the synthesis of defense related compounds such reactive oxygen species and flavonoids (red squares) to stop the spreading of necrotrophic organisms and the death of additional plant cells (gray cells).

In this study we contribute to advance towards understanding the dynamics of grapevine bud necrosis (BN). We report for the first time the putative involvement of endophytic fungi in BN and propose a model that describes how the metabolic interactions between *Vitis vinifera* and endophytic fungi, including *Pyrenochaeta* and *Alternaria alternata* influence BN (Fig. 9). According to this model, sub-optimal environmental conditions such as high light intensity, low photosynthetic levels among others, may impair source to sink ratio of sugar transportation. In this scenario, some organisms living endophytically in the buds can enhance the competition for photoassimilates. When the host plant can no longer support the mutualistic relationships, these endophytes change their biotrophic lifestyle, when they probably colonize between cells and do not harm the host plant, to a necrotrophic lifestyle, when they produce enzymes and toxins to kill the cells of the host plant to obtain nutrients. These changes are mainly due to the increased expression of the *NPS6* gene and an unknown *Mycotymovirys* in *Alternaria alternaria or Pyrenochaeta* sp. during BN. Under these circumstances the salicylic and jasmonic acid signaling pathways are used to trigger defense mechanisms such as the production of ROS, phenolic compounds such as flavonoids and finally programmed cell death. This idea is supported by the enhanced expression of key genes involved in all the processes mentioned above during BN (Fig. 3; Supplementary Figs. 3, 6; Supplementary Tables 1, 2, 5 and 6). The activation of genes related to host programmed cell death would cause the necrotic spots as a defense mechanism to stop the depletion of photoassimilates and the dissemination of the necrotrophic agents.

## Supplementary information

Supplementary Figures.

Supplementary Table 1.

Supplementary Table 2.

Supplementary Table 3.

Supplementary Table 4.

Supplementary Table 5.

Supplementary Table 6.

Supplementary Table 7.

Supplementary Table 8.

Supplementary Table 9.

Supplementary Table 10.
