# Supplementary material for: Transcriptome analyses suggest that changes in fungal endophyte lifestyle could be involved in grapevine bud necrosis
Source: Sci Rep. 2020 Jun 11;10:9514. doi: 10.1038/s41598-020-66500-0 (PMC7290027; doi:10.1038/s41598-020-66500-0)

Supplementary figures

Transcriptome analyses suggest that changes in fungal endophyte lifestyle could be involved in grapevine bud necrosis

Thales Henrique Cherubino Ribeiro[1], Christiane Noronha Fernandes-Brum[1], Claudia Rita de Souza[2], Frederico Alcantara Novelli Dias[2], Osmar De Almeida Junior[3], Murilo de Albuquerque Regina[2], Kellen Kauanne Pimenta de Oliveira[1], Gabriel Lasmar dos Reis[1], Larissa Maia Oliveira[4], Fernanda de Paula Fernandes[2], Laurent Torregrosa[5], Jorge Teodoro de Souza[4], Antonio Chalfun-Junior (ORCID 0000-0002-1794-9429)[1]*

1. Laboratory of Molecular Plant Physiology, Department of Biology, Federal University of Lavras, Lavras, Brazil
2. Technological Center of Grape and Wine Research, Agronomical Research Institute of Minas Gerais, Caldas, Brazil
3. Coordination of Integral Technical Assistance of São Paulo, São Paulo, Brazil
4. Department of Phytopathology, Federal University of Lavras, Lavras, Brazil
5. Montpellier SupAgro, France

***Corresponding author at: Laboratory of Molecular Plant Physiology, Biology Department, Federal University of Lavras (UFLA), s/n - Cx. P. 3037- Minas Gerais, Brazil. Fax: +55-35-3829-1887, chalfunjunior@ufla.br**

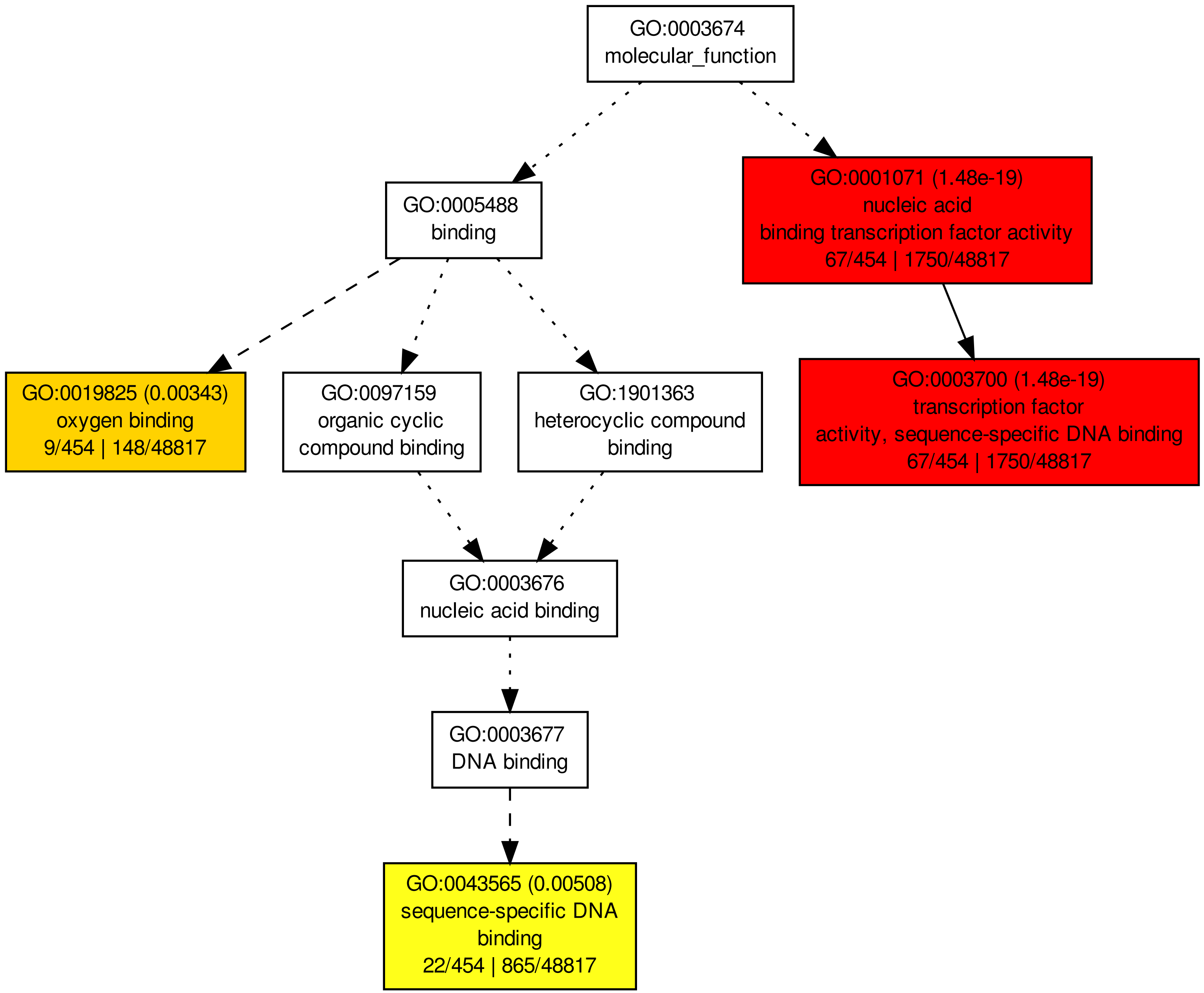
GO:0003674
molecular_function
GO:0005488
binding
GO:0001071 (1.48e-19)
nucleic acid
binding transcription factor activity
67/454 | 1750/48817
GO:0019825 (0.00343)
oxygen binding
9/454 | 148/48817
GO:0097159
organic cyclic
compound binding
GO:1901363
heterocyclic compound
binding
GO:0003700 (1.48e-19)
transcription factor
activity, sequence-specific DNA binding
67/454 | 1750/48817
GO:0003676
nucleic acid binding
GO:0003677
DNA binding
GO:0043565 (0.00508)
sequence-specific DNA
binding
22/454 | 865/48817

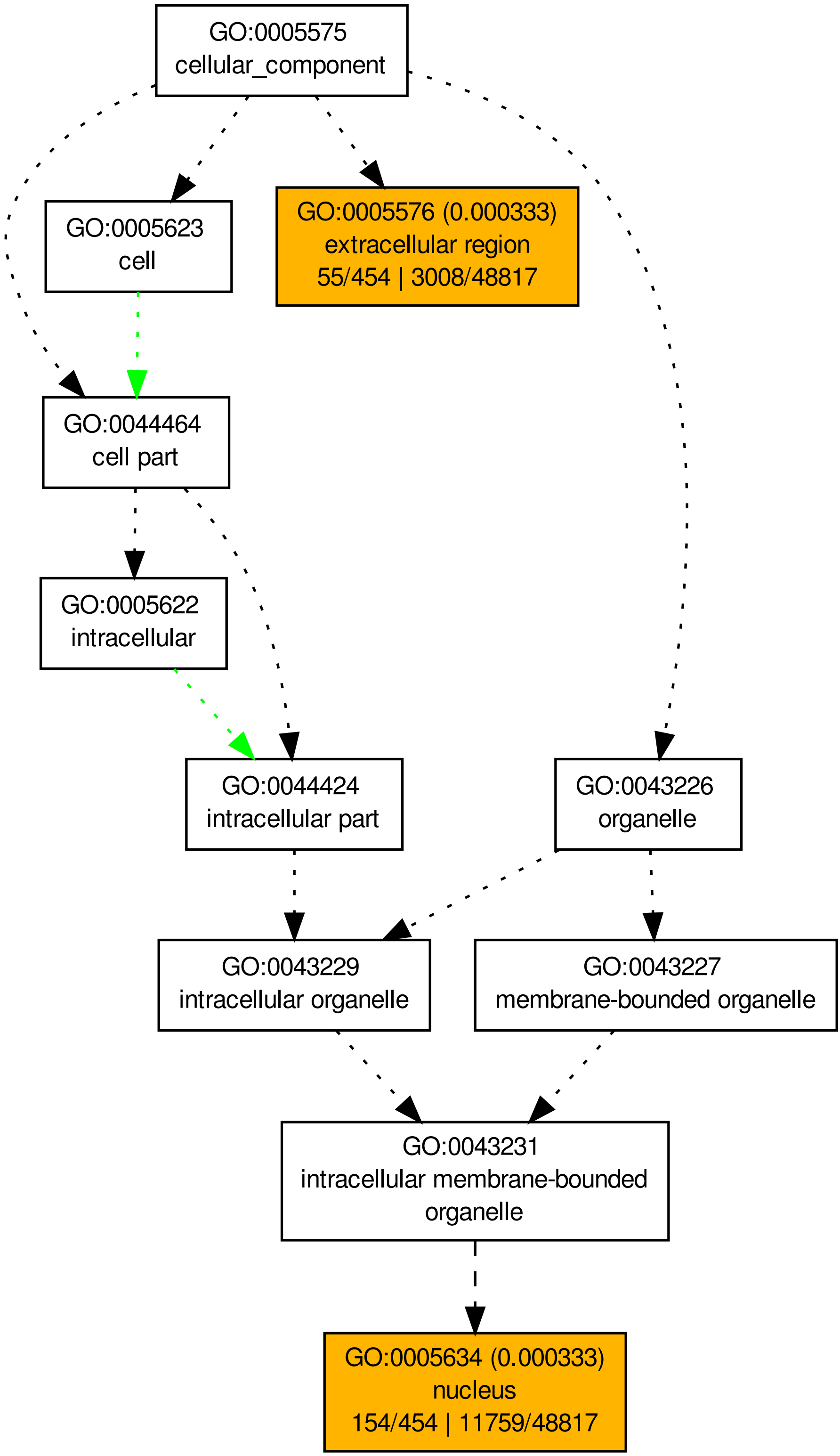

GO:0005575
cellular_component
GO:0005623
cell
GO:0005576 (0.000333)
extracellular region
55/454 | 3008/48817
GO:0044464
cell part
GO:0005622
intracellular
GO:0044424
intracellular part
GO:0043226
organelle
GO:0043229
intracellular organelle
GO:0043227
membrane-bounded organelle
GO:0043231
intracellular membrane-bounded
organelle
GO:0005634 (0.000333)
nucleus
154/454 | 11759/48817

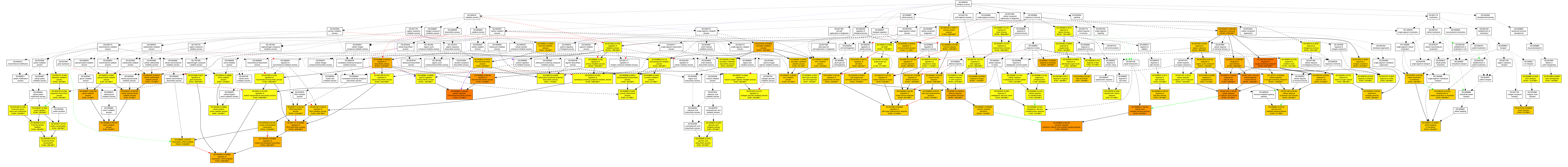

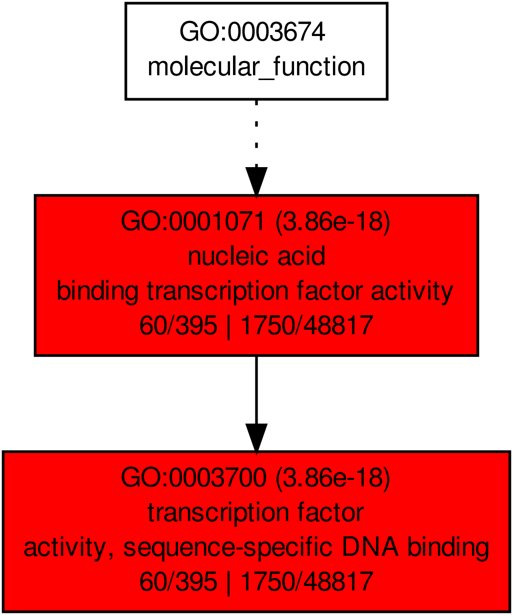
GO:0003674
molecular_function
GO:0001071 (3.86e-18)
nucleic acid
binding transcription factor activity
60/395 | 1750/48817
GO:0003700 (3.86e-18)
transcription factor
activity, sequence-specific DNA binding
60/395 | 1750/48817

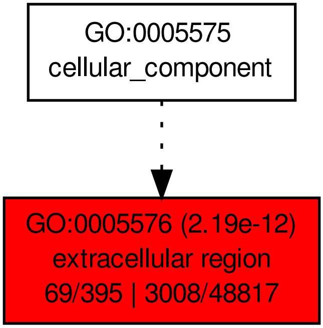
GO:0005575
cellular_component
GO:0005576 (2.19e-12)
extracellular region
69/395 | 3008/48817

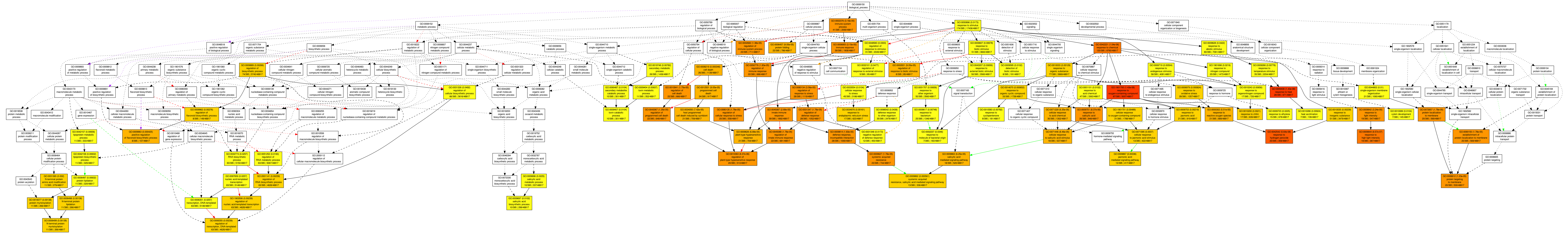

Supplement: Supplementary file 1 — Supplementary Figures. [file 41598_2020_66500_MOESM1_ESM.pdf]
